# Supplementary material for: Splice-Junction-Based Mapping of Alternative Isoforms in the Human Proteome
Source: Cell Rep. Author manuscript; Available in PMC 2020 Jan 15. (PMC6961840; doi:10.1016/j.celrep.2019.11.026)
Supplement: 2 [file NIHMS1546469-supplement-2.zip › DF1/PXD009737/TestisTrypsin/TestisTrypsin_73_MAST4_GSLGGALTDFTTALQVWPCSTQELEGGTFFQTR.pdf]

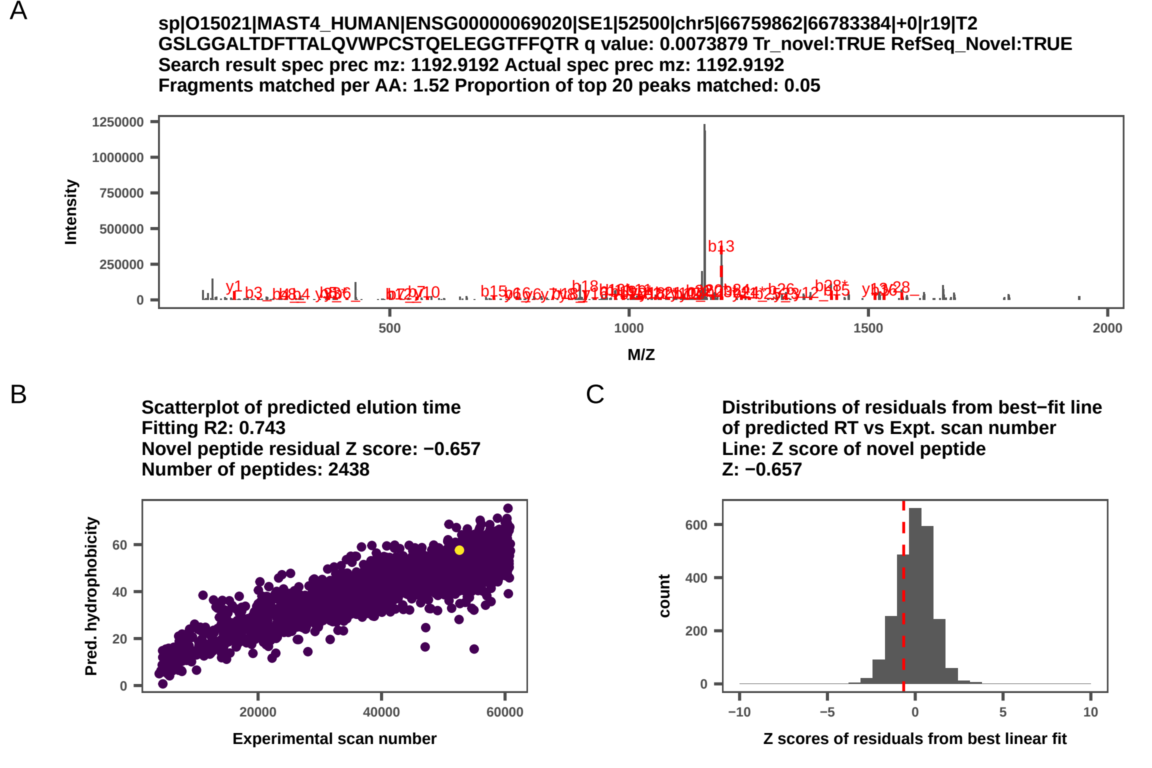
A
sp|O15021|MAST4_HUMAN|ENSG00000069020|SE1|52500|chr5|66759862|66783384|+0|r19|T2
GSLGGALTDFTTALQVWPCSTQELEGGTFFQTR q value: 0.0073879 Tr_novel:TRUE RefSeq_Novel:TRUE
Search result spec prec mz: 1192.9192 Actual spec prec mz: 1192.9192
Fragments matched per AA: 1.52 Proportion of top 20 peaks matched: 0.05
Intensity
1250000
1000000
750000
500000
250000
0
b13
y1
b3
b15
b18
500
1000
1500
2000
M/Z
B
Scatterplot of predicted elution time
Fitting R2: 0.743
Novel peptide residual Z score: −0.657
Number of peptides: 2438
Pred. hydrophobicity
60
40
20
0
20000
40000
60000
Experimental scan number
C
Distributions of residuals from best−fit line
of predicted RT vs Expt. scan number
Line: Z score of novel peptide
Z: −0.657
count
600
400
200
0
−10
−5
0
5
10
Z scores of residuals from best linear fit
